# Reduction in hypoxia‐reoxygenation‐induced myocardial mitochondrial damage with exogenous methane

**DOI:** 10.1111/jcmm.16498

**Published:** 2021-05-04

**Authors:** Dávid Kurszán Jász, Ágnes Lilla Szilágyi, Eszter Tuboly, Bálint Baráth, Anett Roxána Márton, Petra Varga, Gabriella Varga, Dániel Érces, Árpád Mohácsi, Anna Szabó, Renáta Bozó, Kamilla Gömöri, Anikó Görbe, Mihály Boros, Petra Hartmann

**Affiliations:** ^1^ Institute of Surgical Research University of Szeged Szeged Hungary; ^2^ MTA–SZTE Research Group on Photoacoustic Spectroscopy University of Szeged Szeged Hungary; ^3^ Department of Dermatology and Allergology University of Szeged Szeged Hungary; ^4^ Department of Biochemistry University of Szeged Szeged Hungary

**Keywords:** anoxia, cardiomyocytes, complex I, methane, mitochondrial membrane potential, mitochondrial respiration, reoxygenation

## Abstract

Albeit previous experiments suggest potential anti‐inflammatory effect of exogenous methane (CH_4_) in various organs, the mechanism of its bioactivity is not entirely understood. We aimed to investigate the potential mitochondrial effects and the underlying mechanisms of CH_4_ in rat cardiomyocytes and mitochondria under simulated ischaemia/reperfusion (sI/R) conditions. Three‐day‐old cultured cardiomyocytes were treated with 2.2% CH_4_‐artificial air mixture during 2‐hour‐long reoxygenation following 4‐hour‐long anoxia (sI/R and sI/R + CH_4_, n = 6‐6), with normoxic groups serving as controls (SH and SH + CH_4_; n = 6‐6). Mitochondrial functions were investigated with high‐resolution respirometry, and mitochondrial membrane injury was detected by cytochrome c release and apoptotic characteristics by using TUNEL staining. CH_4_ admixture had no effect on complex II (CII)‐linked respiration under normoxia but significantly decreased the complex I (CI)‐linked oxygen consumption. Nevertheless, addition of CH_4_ in the sI/R + CH4 group significantly reduced the respiratory activity of CII in contrast to CI and the CH_4_ treatment diminished mitochondrial H_2_O_2_ production. Substrate‐induced changes to membrane potential were partially preserved by CH_4_, and additionally, cytochrome c release and apoptosis of cardiomyocytes were reduced in the CH_4_‐treated group. In conclusion, the addition of CH_4_ decreases mitochondrial ROS generation via blockade of electron transport at CI and reduces anoxia‐reoxygenation‐induced mitochondrial dysfunction and cardiomyocyte injury in vitro.

## 1 INTRODUCTION

Ischaemic heart disease is a leading cause of death worldwide. The underlying pathophysiology is multifactorial, but mitochondrial dysfunction, is thought to be the common denominator in ischaemia or ischaemia/reperfusion (I/R)‐mediated cardiomyocyte‐damaging events.^1^, ^2^

Methane (CH_4_) forms part of the gaseous environment, which maintains the metabolism within living aerobic cells. Though it is considered to be biologically inert, several studies have demonstrated bioactivity for exogenous CH_4_ in animal models of ischaemia and inflammation,^3^, ^4^, ^5^ and accumulating experimental data suggest that exogenous CH_4_ can influence mammalian energy homeostasis as well.^3^, ^4^, ^5^, ^6^ More importantly, the administration of CH_4_ has improved cardiac function, reduced the level of necroenzymes and prevented myocardial fibrosis and remodelling in acute and chronic rodent models of myocardial infarction.^4^ Whereas these results suggest a causal link between increased CH_4_ input and the hypoxia‐induced oxido‐reductive stress response, the subcellular mechanisms of action are still unclear.

The major goal of this study was to outline a mitochondrial pathway, which explains the bioactivity of CH_4_. In designing our experiments, we took into account that CH_4_ can easily traverse cell membranes and that the molecules move down their concentration gradient into subcellular compartments.^7^ Further, previous findings have demonstrated that CH_4_ treatment can preserve adenosine‐triphosphate (ATP) production after I/R injuries to the liver and eyes.^5^, ^6^ Therefore, these results strengthened the view that the mitochondrion is among the expected intracellular targets of CH_4_ and led us to hypothesize that increased exogenous CH_4_ input can influence the respiratory activity of cardiac mitochondria.^8^

Against this background, we carried out a sequential exploration of the mitochondrial effects of exogenous CH_4_ in normoxic and simulated I/R environments using a high‐resolution respirometry (HRR) system to quantify the electron transport chain (ETC) responses. Ischaemia can impair the mitochondrial respiration and other oxygen‐dependent cellular functions, leading to reversible or irreversible structural damage; therefore, we also detected cell viability and apoptosis of cardiomyocytes as final outcomes.

## 2 MATERIALS AND METHODS

### 2.1 Photoacoustic spectroscopy (PAS) measurement of CH_4_ concentration

The dynamics of the CH_4_ concentration changes were detected by photoacoustic spectroscopy (PAS), as described previously.^9^ Briefly, PAS is a special mode of spectroscopy, which measures optical absorption indirectly via the conversion of absorbed light energy into acoustic waves. The set‐up allows for online measurements of CH_4_ concentrations with a minimum detectable concentration of 0.25 ppm. The CH_4_ concentration in the medium was measured over a period of 120 min, and samples were taken every 2 min.

### 2.2 Cardiomyocyte cell culture

Neonatal rat cardiac myocytes (NRMCs) were isolated, as described previously.^10^ Briefly, 1‐3‐day‐old Wistar rats were sacrificed by cervical dislocation, and the hearts were excised and collected in ice‐cold phosphate‐buffered saline. After the atria were removed, the ventricles were minced with scissors and digested with 0.25% trypsin for 25 min. The cell suspension was centrifuged at 2000 rpm for 15 min at 4°C. Pelleted cells were pre‐plated for 90 min at 37°C to separate the cardiac myocyte‐enriched fraction. Cardiac myocytes were collected and counted in a Burker chamber and plated into 24‐well plates (7 × 10^4^ cells/well) and into 75 cm^2^ flasks (4 × 10^6^ cells/flask). Cells were harvested in Dulbecco's Modified Eagle's growth medium (DMEM) supplemented with a 10% foetal bovine serum (FBS), 1% glutamine and 1% antibiotic/antimycotic solution for 24 hours, and then, the medium was changed to 1% FBS‐containing growth medium to promote the differentiation of the cardiomyocytes. At the end of the three‐day isolation protocol, the phenotype of NRMCs corresponds to that of cardiomyocytes isolated from adult rats. The cardiac myocytes were kept in a normoxic incubator to maintain physiological conditions (37°C, 5% CO_2_ and 95% air).

### 2.3 Isolation of cardiac mitochondria

Adult Sprague Dawley rats were anaesthetized with sodium pentobarbital (45 mg/kg ip) to harvest the heart. The hearts were homogenized with a glass Potter homogenizer, and the mitochondria were isolated with Gnaiger's method.^11^ The isolated mitochondria were suspended in a 2.5 mL mitochondrial respiration medium (MiRo5) for respirometric analysis and were treated as follows: 2 hours normoxia (95% air and 5%CO_2_) or anoxia (100%N_2_) was followed by reoxygenation (with or without CH_4_) for 30 minutes. At the end of the experiments, mitochondrial function was tested.

### 2.4 Experimental protocols

The experiments were performed in two series using either intact NRMCs (Study I) or isolated cardiac mitochondria (Study II). In the first series, three‐day‐old cardiac myocytes were subjected to 4 hours simulated ischaemia (sI). The cells were kept in a hypoxic chamber (37°C, 95% N_2_ and 5% CO_2_), and the culture medium was changed to a hypoxic solution (in mM: NaCl 119, KCl 5.4, MgSO_4_ 1.3, NaH_2_PO_4_ 1.2, 4‐(2‐hydroxyethyl)‐1‐piperazineethanesulfonic acid (HEPES) 5, MgCl_2_ 0.5, CaCl_2_ 0.9, Na lactate 20; bovine serum albumin (BSA) 0.1%, 310 mOsm/L, pH = 6.4). This was followed by a 2h‐reoxygenation period (R) in a culture medium when cells were kept under either normoxic conditions (37°C, artificial air) or in a chamber with normoxic air supplemented with CH_4_ (37°C, 2.2% CH_4_ + artificial air) (the sI/R and sI/R + CH_4_ groups, respectively). Control groups were kept in a normoxic incubator to maintain physiological conditions for 4 hours (normoxic solution containing 125 mmol/L NaCl, 5.4 mmol/L KCl, 1.3 mmol/L MgSO_4_, 1.2 mmol/L NaH_2_PO_4_, 20 mmol/L HEPES, 0.5 mmol/L MgCl_2_, 1 mmol/L CaCl_2_, 15 mmol/L glucose, 5 mmol/L taurine, 2.5 mmol/L creatine‐monohydrate and 0.1% BSA at pH 7.4), which was followed by a 2h‐reoxygenation period in the normoxic incubator with or without CH_4_ supplementation (the normoxia and normoxia + CH_4_ groups). Isolation rounds from the same litters of newborn rats (8‐16 hearts) were performed for each experimental group resulting in 2‐4 flasks of NRMCs (4 × 106 cells/flask) per isolation (n = 6‐6 per group). Data for all individual wells were analysed. At the end of the isolation protocol, the NRMCs were subjected to HRR and cell viability assays (Figure S1).

In the second series, isolated cardiac mitochondria were treated as follows: anoxia was induced using 100% N_2_ persufflation for 2 hours into a 2 mL volume cuvette containing 1 mL respiratory medium and 1 mL airspace. Anoxia was followed by a reoxygenation period (95% air and 5% CO_2_) with or without 2.2% CH_4_ supplementation for 30 minutes (the A/R and A/R + CH_4_ groups, respectively) (n = 12‐16). In the control groups, the mitochondria were kept in normoxic cuvettes (95% air and 5% CO_2_) with or without 2.2% CH_4_ supplementation (the normoxia and normoxia + CH_4_ groups, respectively). Then, the mitochondria were subjected to HRR (Figure S2).

### 2.5 Examination of mitochondrial functions

High‐resolution respirometry with an Oxygraph‐2k (Oroboros Instruments, Innsbruck, Austria) was used to examine the oxygen consumption of the NRMCs and isolated cardiac mitochondria in various mitochondrial metabolic states, mitochondrial hydrogen peroxide (H_2_O_2_) production and changes to mitochondrial membrane potential. The mitochondrial protein content of the samples was determined by Lowry's method.

### 2.6 Coupling control protocol

Before the mitochondrial metabolic states were examined, a cell permeabilization protocol of the NRMCs was applied in the respirometer chamber (Figure S3).

Next, we applied a coupling control protocol to the permeabilized NRMCs. First, endogenous routine respiration was defined without substrates. Then, the cells were permeabilized with digitonin, and the oxidative phosphorylation capacity (OxPhos, State 3) of the NRMCs was measured by adding 10 mmol/L succinate (Succ) and 5 mmol/L ADP substrates. Subsequently, ATP‐independent respiration was measured using 0.5 µmol/L oligomycin (Omy). Maximal mitochondrial respiratory capacity was then measured by titration of 1 µmol/L carbonyl cyanide p‐trifluoromethoxy‐phenyl‐hydrazine (FCCP). Finally, residual oxygen consumption (ROX) was determined by adding 1 μmol/L rotenone (Rot) and 1 μmol/L antimycin‐A (Ama).

### 2.7 Mitochondrial hydrogen peroxide (H_2_O_2_) production

In this series, mitochondrial H_2_O_2_ release as a marker of reactive oxygen species (ROS) (ie superoxide anion) production was monitored fluorimetrically with the Amplex Red/horseradish peroxidase system, whereby Amplex Red (non‐fluorescent) is oxidized to resorufin. H_2_O_2_ production was calibrated with known amounts of H_2_O_2_. In this setup, ROS release was investigated by adding oxidizing substrates (20 mmol/L glutamate, 10 mmol/L malate, 10 mmol/L succinate, 5 mmol/L ADP) to the mitochondria. On isolated mitochondria, the reverse electron transport (RET)‐initiated H_2_O_2_ flux was measured when mitochondria were incubated with 10 mmol/L succinate; it was then blocked by the addition of 1 µmol/L rotenone. The residual oxygen consumption was estimated after addition of 1 μmol/L antimycin‐A (inhibitor of CIII) to exclude the effects of oxidative side reactions. Then, free radical leak was also determined as the percentage of oxygen consumption diverted to the production of H_2_O_2_ in State 3.

### 2.8 Mitochondrial membrane potential

Mitochondrial membrane potential was measured fluorimetrically using the fluorophore safranin. First, we added 1 µmol/L Rot, 10 mmol/L succinate and 1 µmol/L FCCP. Finally, ROX was determined by adding 1 μmol/L antimycin‐A (Ama). Extramitochondrial Ca^2+^ movement (as it indicates strong correlation with membrane potential^12^) was examined with the use of blue fluorescence sensor of HRR (excitation 465 nm; gain for sensor: 1000 and polarization voltage: 500 mV) (Figure S4).

### 2.9 Detection of cytochrome c oxidase activity

Cytochrome c oxidase activity was calculated via the time‐dependent oxidation of cytochrome c at 550 nm, as described previously. Briefly, a cytochrome c stock solution was freshly prepared by dissolving 10.6 mg cytochrome c (Sigma‐Aldrich, Budapest, Hungary) in 20 mL distilled water. The cytochrome c was then reduced by adding 50 µL 0.1 mol/L sodium dithionite, with the absorbance of the solution determined at 550 nm; the photometer was calibrated to this level. Heart samples were homogenized with a Potter grinder in 10× ice‐cold Miro5 medium and then centrifuged at 800 *g* for 5 minutes at 4°C. 50 μL supernatant was added to 2.5 mL cytochrome c stock solution, and the decrease in optical density at 550 nm was measured spectrophotometrically during 1 minute intervals at 0, 30 and 60 minutes.

### 2.10 TUNEL and DAPI staining

Apoptosis of the NRMCs was detected with the TUNEL method. First, the cell number was detected, and then, a cytocentrifuge (6 minutes, 600 rpm, 50 000 cells/slide) was used to create cytospin samples. Samples (n = 6 each) were analysed for apoptotic cell staining with In Situ Cell Death Detection Kit TMR red (Roche, Cat. No. 12 156 792 910). The cytospin samples were fixed in 4% paraformaldehyde for 60 minutes and then permeabilized on ice for 2 minutes in 0.1% Triton X‐100 in 0.1% sodium citrate. We used one part enzyme solution and nine parts label solution for the TUNEL reaction mixture according to the manufacturer's instructions. The cytospin samples were incubated in the dark for 60 minutes at 37°C in a humidified atmosphere, followed by DAPI staining (Sigma‐Aldrich, Cat. No. 10236276001). For each experimental series, one negative control (incubated only with the label solution) and one positive control (digested with DNase I (Quiagen, Cat. No. 79254) together with the TUNEL reaction mixture), and three normal (only with the TUNEL mixture) samples were used. Three pictures were taken of each sample (negative control, positive control and three normal samples each) in each experimental series with a Zeiss AxioImager.Z1 microscope at 20× magnification. The number of apoptotic cells per field of view (524.19 μm × 524.19 μm) was determined with Image J 1.47 software.

### 2.11 Cell viability assay and lactate dehydrogenase (LDH) release

The NRMCs were incubated with 1 μmol/L calcein acetoxymethyl ester (Sigma‐Aldrich, Cat. No. 56496 calcein‐AM, Sigma, St. Louis, MO) dissolved in dimethyl sulfoxide at room temperature for 30 minutes to assess cell viability. Fluorescence intensity was measured with a fluorescence plate reader (Fluostar Optima, BMG Labtech, Ortenberg, Germany). Cell viability was compared with that of vehicle control. Cytotoxicity was also measured with the level of LDH released from the damaged cells into the medium culture with the available commercial LDH activity assay kit (Sigma‐Aldrich, Cat. No. MAK066 Sigma‐Aldrich, Budapest, Hungary) according to the manufacturer's instructions.

## 3 RESULTS

### 3.1 CH_4_ concentrations

**FIGURE 1:**
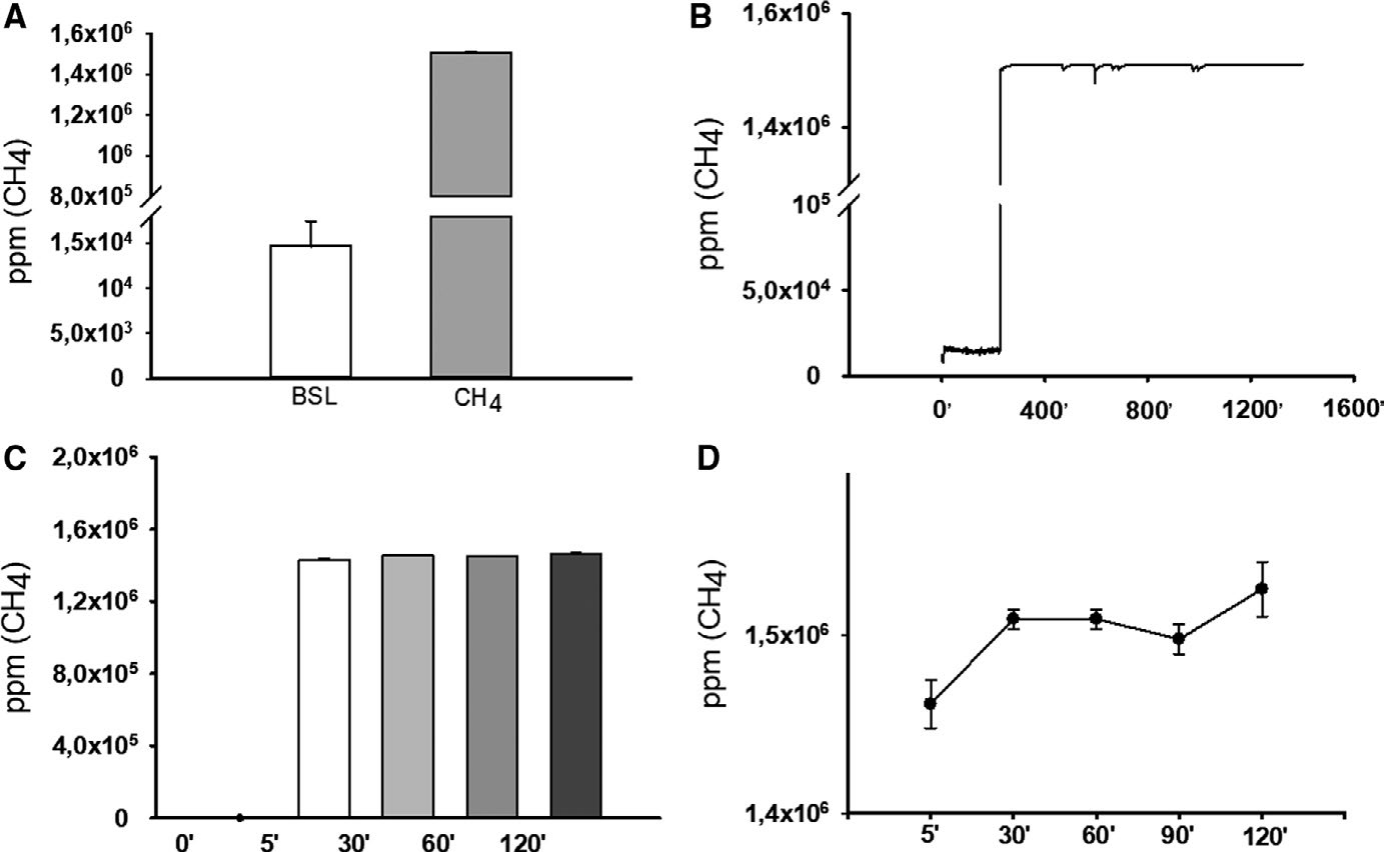
CH4 concentrations measured by photoacoustic spectroscopy (PAS). A, The CH_4_ concentration in the airspace of the incubator (white column: baseline/background concentration; grey column: concentration under the persufflation with a 2.2% CH_4_–artificial air mixture). B, Representative record of CH_4_ measurement in the airspace of the incubator. C, The change in the dissolved CH_4_ concentration of the cell culture medium under the persufflation with the 2.2% CH_4_–artificial air mixture. D, The CH_4_ concentration of the medium shown in a narrower range. Data are presented as means ± SEM. #*P* < .05 vs. baseline/background CH4 concentration (one‐way ANOVA, Tukey's test)

The background CH_4_ concentration in the airspace of the incubation chambers was 1.46.10^4^ ± 94.95 ppm, and a rapid two orders of increase (to 1.5.10^6^ ± 58.12 ppm) was detected after the start of persufflation with a 2.2% CH_4_‐artificial air mixture. This concentration was steadily maintained during the 2 hours reoxygenation period (Figure 1A,B). The dissolved CH_4_ concentration was 1.46.10^6^ ± 76 381 ppm in the cell culture medium and 1.41.10^6^ ± 61 314 ppm in the MiRo 5 respiration medium 5 minutes following CH_4_ persufflation (Figure 1C,D).

### 3.2 Effect of CH_4_ on the mitochondrial functions of the neonatal rat cardiomyocytes (NRMCs)

**FIGURE 2:**
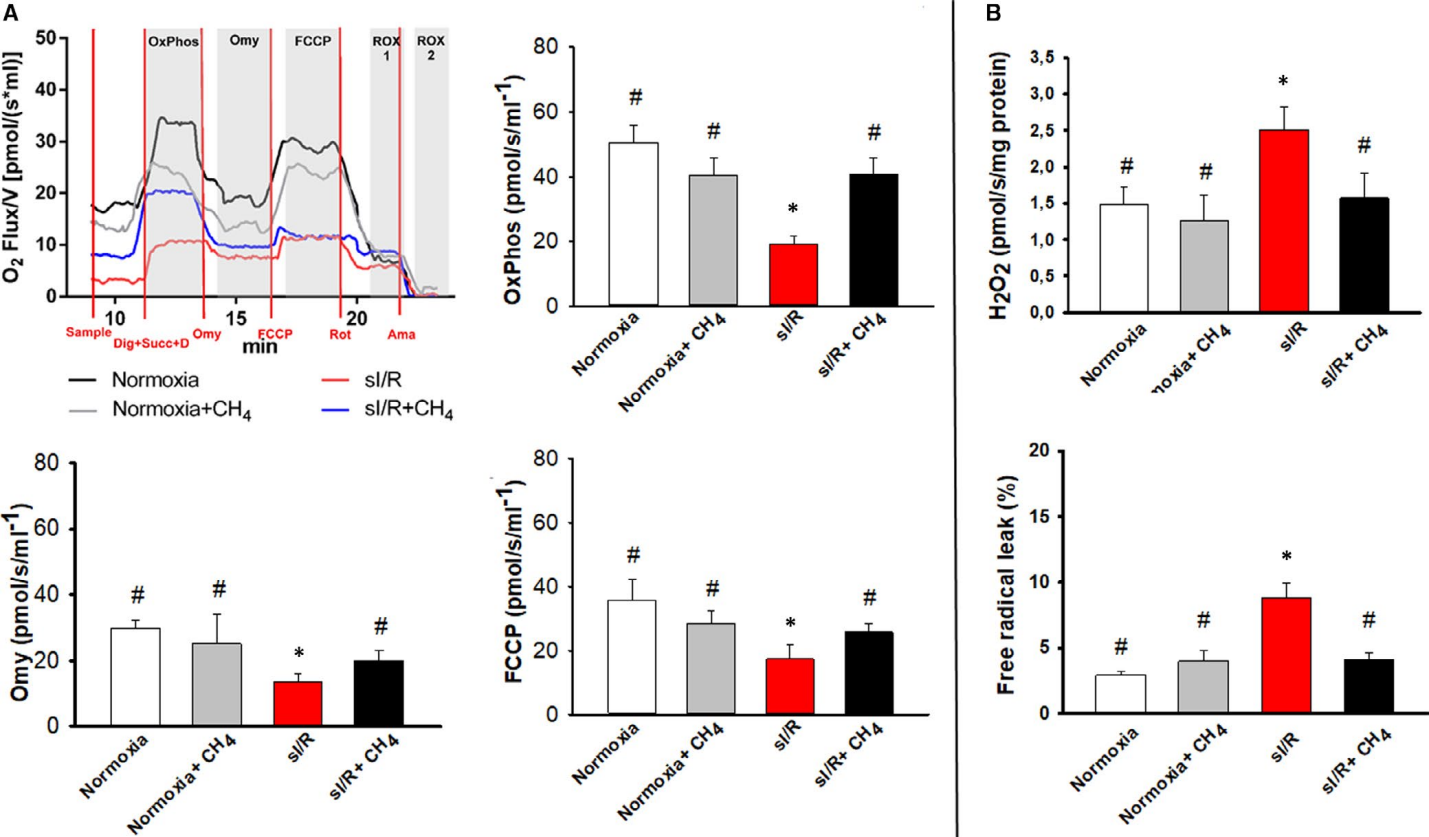
The effect of CH_4_ incubation on the neonatal rat cardiomyocytes (NRMCs). A, The oxygen consumption of the NRMCs (pmol/s/mL^−1^). The upper left‐hand chart demonstrates representative records of mitochondrial oxygen consumption measured by HRR. The upper right‐hand chart shows the oxidative phosphorylation (OxPhos), the lower left‐hand chart presents the oligomycin leak (Omy), and the lower right‐hand chart displays the maximum respiratory capacity (FCCP). B, Hydrogen peroxide (H_2_O_2_) production of the NRMCs. The upper chart represents the mitochondrial H_2_O_2_ production of the NRMCs in pmol/s/mg protein. The lower chart shows the free radical leak expressed as the percentage of oxygen consumption diverted by the H_2_O_2_ production in State 3. sI/R: simulated ischemia/reperfusion; CH_4_: methane; Dig + Succ + D: 1 μL digitonin + 10 mmol/L succinate + 5 mmol/L ADP; Omy: 0.5 μmol/L oligomycin; FCCP: 1 μmol/L carbonyl cyanide p‐trifluoro‐methoxyphenyl hydrazine; Rot: 1 μmol/L rotenone; Ama: 1 μmol/L antimycin‐A; OxPhos: oxidative phosphorylation; ROX: residual oxygen consumption; white columns: normoxia group; grey columns: normoxia + CH_4_ group; red columns: sI/R group; black columns: sI/R + CH_4_ group. Data are presented as means ± SEM. *P < .05 vs. normoxia; #*P* < .05 vs. sI/R (one‐way ANOVA, Tukey's test)

The coupling control protocol provides an opportunity to analyse the leak respiration of the mitochondria. As a result, significantly lower OxPhos was measured in the sI/R group in comparison with the normoxia group (19.17 ± 9.37 pmol/s*mL vs. 50.51 ± 12.87 pmol/s*mL; *P* < .001) (Figure 2A). CH_4_ treatment in the sI/R + CH_4_ group significantly enhanced oxygen consumption (to 40.88 ± 15.08 pmol/s*mL; *P* = .004) (Figure 2A). The leak respiration decreased during sI/R (13.54 ± 2.66 pmol/s*mL; *P* < .001); however, it was ameliorated as a result of CH_4_ administration in the sI/R + CH_4_ group (19.94 ± 3.15 pmol/s*mL; *P* < .001) (Figure 2A). The sI/R significantly lowered the maximum respiratory capacity in comparison with the normoxia group (17.35 ± 4.46 pmol/s*mL vs. 35.72 ± 6.55 pmol/s*mL; *P* < .001) (Figure 2A). CH_4_ treatment had no effect on the maximum respiratory capacity during sI/R (18.41 ± 2.99 pmol/s*mL; *P* = .986) (Figure 2A). Flux values in different states were corrected for ROX (data not shown). There was a significant rise in mitochondrial H_2_O_2_ levels in the I/R group as compared to the normoxia group. Incubation with CH_4_ during reoxygenation lowered the amount of H_2_O_2_ production (Figure 2B). The free radical leak was increased in the sI/R group; however, this rise was ameliorated as a result of CH_4_ administration in the sI/R + CH_4_ group (8.74 ± 3.85 vs. 4.09 ± 1.57; *P* < .05) (Figure 2B). The CH_4_ incubation had no effect on the free radical leak in the normoxia + CH_4_ group compared with the normoxia group (3.97 ± 2.80 vs. 2.91 ± 0.64; *P* > .05) (Figure 2B).

### 3.3 Apoptosis, cytochrome c oxidase activity and viability of NRMCs

**FIGURE 3:**
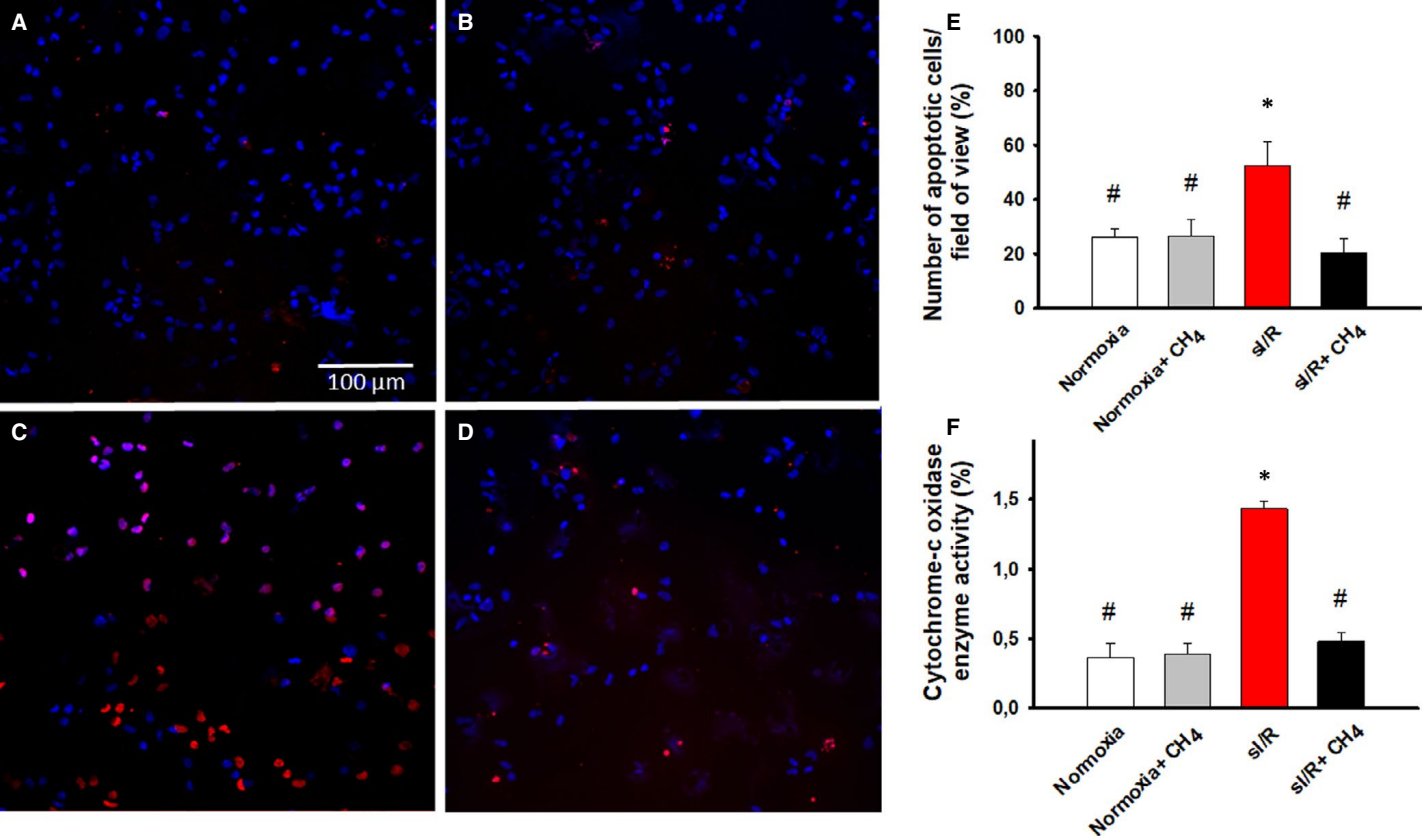
Cell apoptosis and cytochrome c oxidase activity. A, Normoxia group. B, Normoxia+CH4 group. C, sI/R group. D, sI/R + CH_4_ group. E, The number of apoptotic cells (%). F, Cytochrome c oxidase activity (%). White column: normoxia group; grey column: normoxia + CH_4_ group; red column: sI/R group; black column: sI/R + CH_4_ group. Data are presented as means ± SEM. *P < .05 vs. normoxia; #*P* < .05 vs. sI/R (one‐way ANOVA, Tukey's test)

Neonatal rat cardiac myocytes were marked with TUNEL/DAPI staining to examine the presence of apoptosis. As expected, few TUNEL‐positive cells were observed in the normoxia and normoxia + CH_4_ groups (26 ± 9% and 26.3 ± 12% of cells, respectively; *P* = 1.00) (Figure 3A–B,E). sI/R was accompanied by an increased TUNEL positivity (sI/R: 52.4 ± 12% of cells) (Figure 3C,E), which was diminished as a result of CH_4_ incubation (sI/R + CH_4_: 20.1 ± 16.4 of cells; *P* = .01) (Figure 3D,E). The mitochondrial cytochrome c oxidase activity was determined with a spectrophotometric analysis. Remodelling the mitochondrial membrane during I/R results in cytochrome c release to the cytosol; therefore, this event can be considered an indicator of mitochondrial membrane damage. In the normoxia + CH_4_ group, the enzyme activity did not change in response to CH_4_ incubation as compared to the normoxia group (0.39 ± 0.17 vs. 0.37 ± 0.24; *P* = .992) (Figure 3F). In contrast, sI/R was accompanied by increased cytochrome c oxidase activity (1.43 ± 0.13; *P* < .001) (Figure 3F), which was diminished as a result of CH_4_ incubation (0.48 ± 0.15; *P* < .001) (Figure 3F).

**FIGURE 4:**
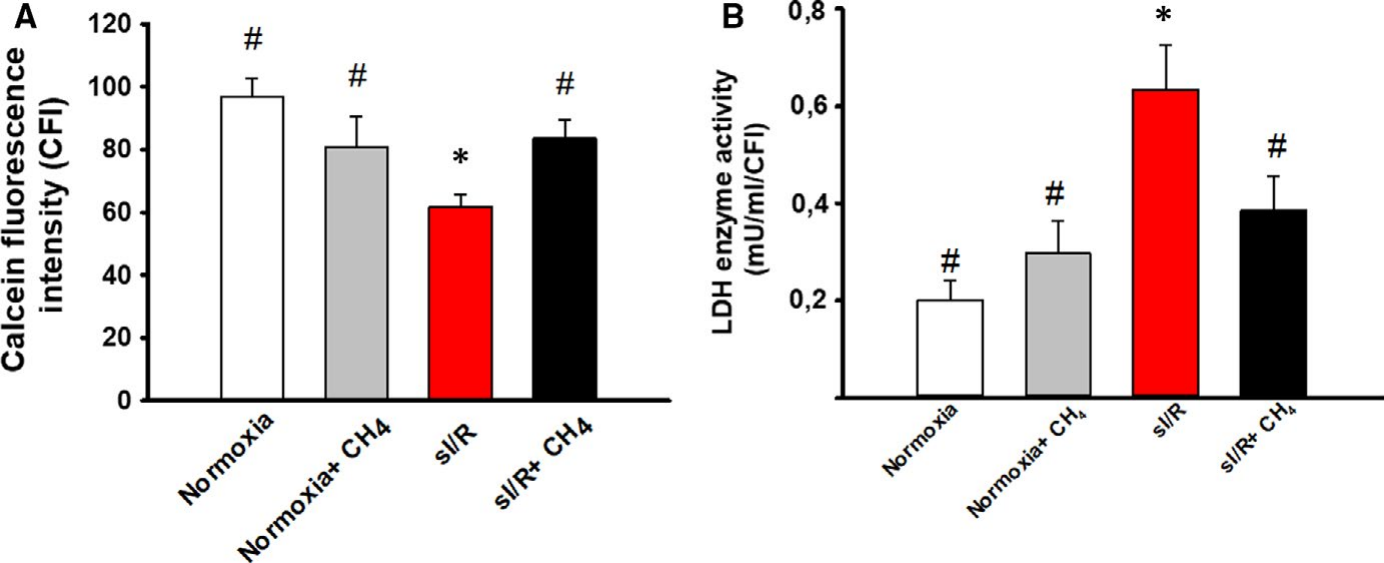
Cell viability of the NRMCs. A, Representative image of calcein assay of cell viability. B, The number of living cells. C, Lactate dehydrogenase (LDH) enzyme activity. White column: normoxia group; grey column: normoxia + CH_4_ group; red column: sI/R group; black column: sI/R + CH_4_ group. Data are presented as means ± SEM. *P < .05 vs. normoxia; #*P* < .05 vs. sI/R (one‐way ANOVA, Tukey's test)

Cardiomyocyte viability was determined with a calcein‐based viability assay (Figure 4A). During the measurements, calcein passed through the cell membrane and hydrolysed to green fluorescent calcein because of the endogenous esterases in the living cells. Compared with the normoxia group, the CH_4_ treatment led to a small drop in viability in the normoxia+CH_4_ group (93.48 ± 14.32 vs. 83.89 ± 12.91; *P* = .891) (Figure 4B). Because of sI/R, the number of living cells decreased, a change shown by the significantly reduced calcein fluorescent intensity (61.74 ± 9.76; *P* = .041). Cell death because of sI/R was prevented with the CH_4_ treatment in the sI/R + CH_4_ group (86.63 ± 12.03; *P* = .003) (Figure 4B).

In the case of the LDH activity assay, there was no difference in this parameter between the two normoxic groups (0.22 ± 0.12 vs. 0.35 ± 0.11; *P* = .15) (Figure 4C). The LDH concentration was significantly lower in the sI/R + CH_4_ group than in the sI/R group (0.42 ± 0.10 vs. 0.68 ± 0.12; *P* = .041) (Figure 4C).

### 3.4 Effects of CH_4_ on isolated cardiac mitochondria

**FIGURE 5:**
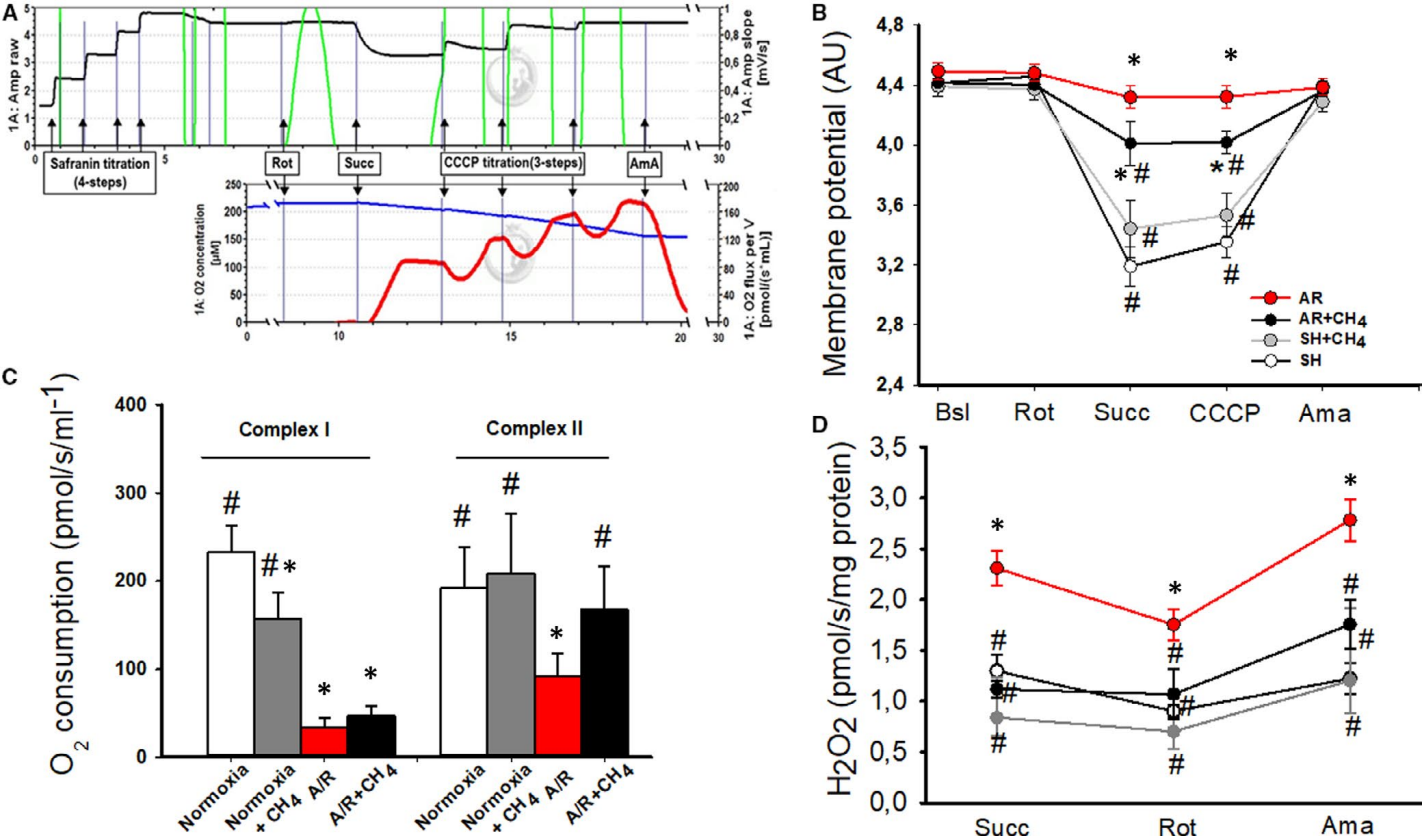
The effect of CH_4_ on isolated cardiac mitochondria. The upper left‐hand chart demonstrates representative records of mitochondrial membrane potential measured fluorimetrically by HRR. The continuous black line indicates changes in membrane potential; in parallel, the red line signifies the substrate‐fuelled respiration. The upper right‐hand chart presents changes in membrane potential in the experimental groups. The A/R group is labelled with a red line, the A/R + CH_4_ group with a black line, and the normoxia and normoxia + CH_4_ groups with pale and dark grey lines, respectively. The lower left‐hand chart shows complex I and II‐driven mitochondrial oxygen consumption. The lower right‐hand chart demonstrates H_2_O_2_ production in the case of reverse electron transfer (RET)

Changes to mitochondrial membrane potential have been characterized by means of the potential‐sensitive fluorophore safranin. Substrates of respiratory complexes induced a significant hyperpolarization in the mitochondrial membrane under normoxic conditions (Figure 5A). In contrast, hyperpolarization was eliminated in the AR group. Substrate‐induced changes in membrane potential were partially preserved by CH_4_ supplementation (Figure 5A). CH_4_ applied during the anoxic period lowered the amount of H_2_O_2_ production in leak states (Figure 5B). In terms of oxygen consumption, we investigated complex I and succinate‐semialdehyde dehydrogenase (complex II)‐linked respiration separately. CH_4_ significantly decreased the oxygen consumption of complex I, whereas it had no effect on complex II‐linked respiration under normoxic conditions. In contrast, CH_4_ treatment in the sI/R + CH_4_ group significantly improved the oxygen consumption of complex II compared with complex I (Figure 5B).

## 4 DISCUSSION

Our primary aim was to outline a possible mechanism linked to the in vivo biological efficacy of CH_4_. The expected mitochondrial effects of CH_4_ have been characterized by HRR, and we have shown that the administration of CH_4_ reduces the sI/R‐related mitochondrial ETC disturbance and mitigates subsequent apoptotic consequences. Of importance, CH_4_ preserved mitochondrial membrane potential (a marker of the integrity of the inner mitochondrial membrane) and decreased cytochrome c release (a sign of the integrity of the outer mitochondrial membrane) as well.

According to current knowledge, CH_4_ is not involved in catabolic or metabolic biochemical processes in the eukaryotic cell. Interestingly, in a pre‐clinical model of myocardial infarction, CH_4_ treatment significantly improved the cardiac function and reduced the apoptosis of cardiomyocytes.^4^ The anti‐apoptotic and anti‐oxidative effects of CH_4_ have been demonstrated in other I/R settings as well.^3^, ^4^, ^5^ These data all suggest that the underlying mechanism of action is intimately connected with the mitochondrial functions.

Excessive oxidative stress is a major component of sI/R, and the mitochondrial ETC is a dominant source of ROS generation. Likewise, the majority of superoxide production is linked to complex I early in reperfusion.^13^, ^14^, ^15^, ^16^ This notion has been supported by studies showing that ischaemic preconditioning or pre‐treatment with reversible complex I inhibitors can limit ROS generation and cardiac IR injury.^14^, ^16^ In this primary mitochondrial model of cardiac IR injury, we investigated mitochondrial coupling states with HRR. We tracked mitochondrial ROS production with HRR by using the fluorescent dye Amplex Red, and, in parallel, the mitochondrial membrane potential was measured using the potential‐sensitive fluorescence dye safranin. The respiratory activity of complex I remained stunned in the reoxygenation phase in line with the overwhelming ROS production. In the presence of CH_4_, the complex I‐linked respiration decreased in both control and simulated ischaemia‐damaged mitochondria, but there were no changes in the presence of rotenone, an irreversible complex I inhibitor. This suggests that CH_4_ treatment reduced ROS generation via a partial blockade of electron transport in complex I. It should be added that two sites for superoxide production have recently been explored on respiratory complex I, the ubiquinone (Q)‐binding and flavin sites.^15^ The superoxide production at the flavin site is linked to the forward electron transport, and its rate depends on the reduction state of the matrix nicotinamide adenine dinucleotide (NAD) pool. More importantly, the Q‐binding site produces superoxide at much higher rates than the flavin site, driven by the reverse electron transport (RET) from complex II into complex I during reoxygenation.^17^ In isolated cardiac mitochondria, the underlying mechanism of RET is the accumulation of succinate during hypoxia and its subsequent rapid oxidation at reoxygenation in the presence of a high membrane potential.^18^ Rotenone, an irreversible inhibitor of electron transport at the Q‐binding site has been demonstrated to exert cardioprotection by decreasing RET in the early phase of reperfusion.^14^ Based on our results, the active site of CH_4_ is, as in the case of rotenone, distal to the flavin site, because it enhances mitochondrial ROS generation when the electrons enter complex I from NADH, but it inhibits ROS generation by RET from complex II. Large membrane potential is a prerequisite to drive the electrons against the gradient of redox potentials from complex II into complex I. It has been demonstrated in isolated mitochondria that only a 5% reduction in mitochondrial membrane potential will reduce peroxide production by 95%.^19^ Any manipulation of the RET pathway could potentially influence the end outcome of ROS production, even by lowering the driving hyperpolarization of the mitochondrial membrane potential. In our system, the addition of normoxic CH_4_ slightly decreased the substrate‐induced hyperpolarization in control mitochondria, in contrast to the preservative effect seen in the case of the anoxia‐damaged membrane.

Next, the role of complex I and complex II in the post‐anoxic cardiac mitochondrial respiration was addressed in more detail. As a result of CH_4_ treatment, respiration was inhibited when glutamate + malate was used as a complex I substrate but not with succinate as a complex II substrate. This finding suggested that the addition of CH_4_ resulted in a decreased electron flux through complex I but did not alter the succinate oxidation through complex II. Based on these findings, the drop in net ROS production from mitochondria with preserved succinate oxidation in the presence of CH_4_ is most likely directly related to the inhibition of complex I. However, CH_4_ appears to provide a blockade of electron transport in complex I in contrast to a complete blockade of oxidative metabolism during reoxygenation.

**FIGURE 6:**
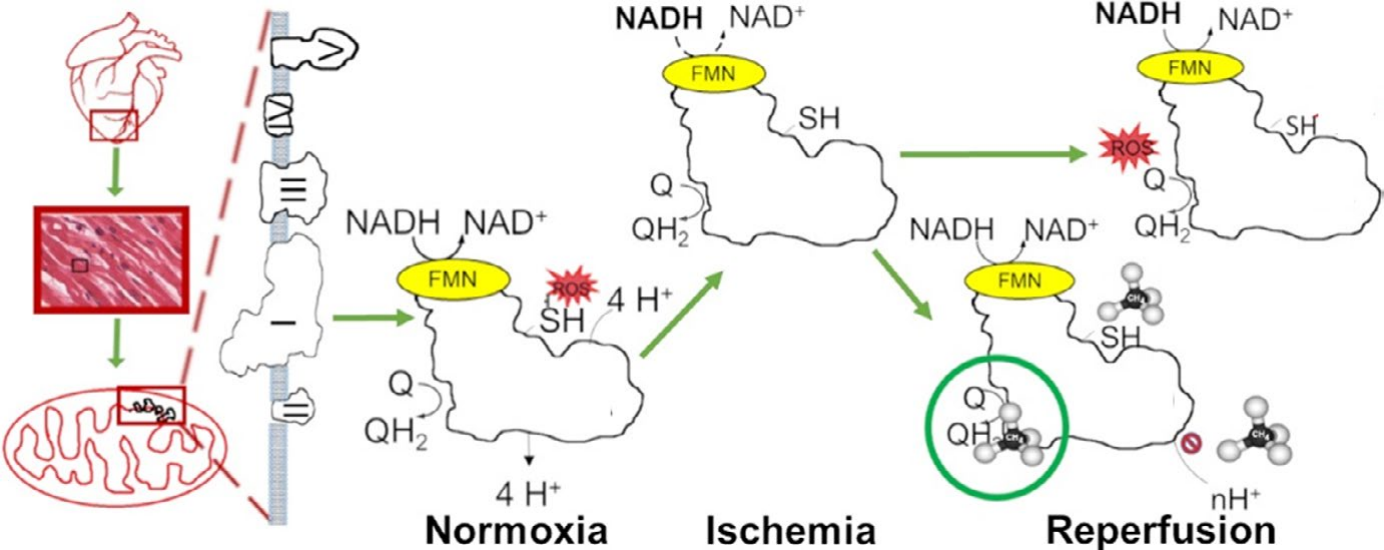
The effects of CH_4_ on complex I. The mechanism of protection likely included a blockade of the electron transport in complex I and decreased ROS generation. Reversible deactivation of mitochondrial complex I is an intrinsic mechanism, which provides a fast response of the mitochondrial respiratory chain to oxygen deprivation. However, subsequent reoxygenation leads to ROS generation due to the rapid burst of respiration. Under normoxic conditions, a high level of nicotinamide adenine dinucleotide hydride (NADH) can drive forward electron flow with superoxide generation at the flavin mononucleotide moiety located near the NADH binding subunit. During reoxygenation, reverse electron flow driven by a reduced ubiquinone (ubiquinol) pool and high proton motive force can generate ROS when electrons flow back from ubiquinol to Complex I. CH_4_ treatment restricts the forward electron transfer within complex I in control mitochondria while effectively inhibiting RET in post‐ischaemic mitochondria

Mitochondrial complex I has two conformations with different catalytic activities: an active state (A) and a deactivated state (D), which are present in A/D equilibrium at a ratio of 9:7 under physiological conditions.^20^ The A/D transition occurs during ischaemia/anoxia as an intrinsic mechanism, which produces a rapid response of the mitochondrial ETC to oxygen deprivation.^21^ Modulating factors of the A/D transition include the availability of oxygen, the reduced NAD pool in the matrix, the temperature and the pH. The physiological role of the accumulation of the D‐form in anoxia is most probably to protect mitochondria from ROS generation because of the rapid burst of respiration following reoxygenation.^13^, ^22^ The transient preservation of complex I in the D‐form was implemented as a successful strategy against reperfusion injury in the post‐ischaemic brain and heart.^13^, ^23^, ^24^ Though the entire NAD pool is reduced under ischaemic/anoxic conditions, the nucleotide at the flavin site could be rapidly decreased by the RET directly from ubiquinol at the beginning of reoxygenation. However, the D‐form of the enzyme is unable to catalyse the RET, and therefore, de‐activation may act as a protective valve by preventing reduction in the enzyme from downstream. Both the D‐form of complex I and the rotenone‐inhibited enzyme have restricted access to the quinone‐binding pocket near the iron‐sulphur cluster.^25^ The functional outcome of this is, on the one hand, similar to the blockade of forward electron transfer within the A‐form by a molecule of rotenone‐like inhibitor bound at the quinone site. On the other hand, the RET occurring in the D‐form of the enzyme can also be restricted because of ubiquinol onto the iron‐sulphur cluster, thereby eliminating the ROS formation.^21^, ^26^ (Figure 6).

In this study, CH_4_ treatment restricted the forward electron transfer within complex I in control mitochondria whereas effectively restricting RET in post‐anoxic mitochondria. It can be concluded that besides other mitochondrial sites, interactions with complex I certainly occupies a key position in the protective mechanism of CH_4_ treatment against sI/R injury. It is likely that this action includes conformational changes in respiratory complex I rather than direct interaction with a membrane‐associated binding site. Earlier notions about the molecular mechanism by which CH_4_ exerts a non‐specific action were linked to the physical properties of the molecule. Hydrocarbon gases may modulate the structure and function of biological membranes, which has been demonstrated in lipid bilayer models in vitro ^27^, ^28^ and in animal models in vivo.^29^, ^30^ As the smallest hydrocarbon molecule, CH_4_ may interact with the cell membrane, leading to haemolysis in erythrocytes in a concentration‐dependent manner.^27^ In our study, a non‐specific action of CH_4_ was demonstrated by increased OxPhos capacity and ameliorated leak respiration in the sI/R + CH_4_ group, linked to the preserved membrane integrity and electron transfer capacity of mitochondria. It should be added that further, targeted in vitro studies may reveal further insights (eg on the involvement of ATP synthase), but this was beyond the scope of the current protocol. Conformational changes ranging from localized motions of side chains to global structural changes are required for small molecules, even gases, to gain access to their target binding site.^31^ Therefore, research on this patient has shifted significantly towards the interaction of gases and proteins with membrane‐mediated conformational change. Typical examples include anaesthetic gases, which have previously been known to exert their effect through the disruption of the membrane, yet accumulate and bind to multiple modulation sites of cellular membrane‐embedded ion channels. Isoflurane and barbiturates have been identified to partition first in the lipid membrane and then bind to the transmembrane domain of the nicotinic acetylcholine receptor.^32^, ^33^ In line with this, halothane has shown demonstrable effects on acetylcholine‐activated ion channel kinetics through its conformational changes.^34^

## 5 CONCLUSION

We have presented our first data on the effects of CH_4_ on transient anoxia‐induced respiratory changes in cardiomyocyte cultures. Evidence suggests that CH_4_ treatment decreases myocyte injury through reduced ROS generation via blockade of electron transport in complex I and improved inner mitochondrial membrane integrity. Further in vivo studies are needed to investigate whether the administration of CH_4_ would provide a way to attenuate the potentially harmful mitochondrial consequences of hypoxia‐reoxygenation insults.

## CONFLICT OF INTEREST

The authors declare that they have no competing interests.

## AUTHOR CONTRIBUTIONS

**Kurszán Jász:** Conceptualization (equal); data curation (lead); formal analysis (equal); investigation (lead); methodology (lead); project administration (equal); validation (equal); visualization (lead); writing‐original draft (lead). **Ágnes Lilla Szilágyi:** Conceptualization (equal); data curation (equal); formal analysis (equal); funding acquisition (equal); investigation (equal); methodology (equal); project administration (equal); writing‐original draft (equal). **Eszter Tuboly:** Investigation (supporting); methodology (equal); project administration (equal); software (supporting). **Bálint Baráth:** Conceptualization (equal); data curation (equal); investigation (equal); methodology (equal); project administration (equal); software (supporting); supervision (supporting); writing‐original draft (supporting). **Anett Roxána Márton:** Data curation (equal); investigation (equal); methodology (equal); project administration (equal); visualization (lead); writing‐original draft (equal). **Petra Varga:** Conceptualization (supporting); investigation (supporting); project administration (supporting); writing‐original draft (supporting). **Gabriella Varga:** Resources (supporting); software (supporting); supervision (supporting). **Dániel Érces:** Resources (supporting); software (supporting); supervision (supporting). **Árpád Mohácsi:** Investigation (equal); methodology (equal); project administration (equal); resources (equal); software (equal); supervision (supporting). **Anna Szabó:** Data curation (equal); resources (equal); software (equal). **Renáta Bozó:** Formal analysis (equal); investigation (lead); methodology (lead); project administration (equal); resources (lead); validation (equal); visualization (lead). **Kamilla Gömöri:** Conceptualization (equal); investigation (equal); methodology (equal); project administration (equal); validation (equal). **Anikó Görbe:** Conceptualization (equal); funding acquisition (equal); resources (equal); software (equal); supervision (equal); validation (equal); visualization (equal). **Mihály Boros:** Conceptualization (equal); resources (equal); software (equal); supervision (equal); validation (equal); writing‐original draft (equal); writing‐review and editing (equal). **Petra Hartmann:** Conceptualization (supporting); data curation (supporting); methodology (supporting); project administration (supporting); resources (lead); supervision (lead); validation (lead); writing‐original draft (supporting); writing‐review and editing (equal).

## ETHICAL APPROVAL

The experimental protocol was in accordance with EU Directive 2010/63 for the protection of animals used for scientific purposes, and it was approved by the National Scientific Ethics Committee on Animal Experimentation (National Competent Authority) under licence number V./148/2013. This study also complied with the criteria in the US National Institutes of Health Guidelines for the Care and Use of Laboratory Animals.

## CONSENT FOR PUBLICATION

Not applicable.

## Supporting information

Fig S1

Fig S2

Fig S3

Fig S4

## Data Availability

The data are all presented in the manuscript.
