# Supplementary figures and images for: Reduction in hypoxia‐reoxygenation‐induced myocardial mitochondrial damage with exogenous methane
Source: J Cell Mol Med. 2021 May 4;25(11):5113–23. doi: 10.1111/jcmm.16498 (PMC8178286; doi:10.1111/jcmm.16498)

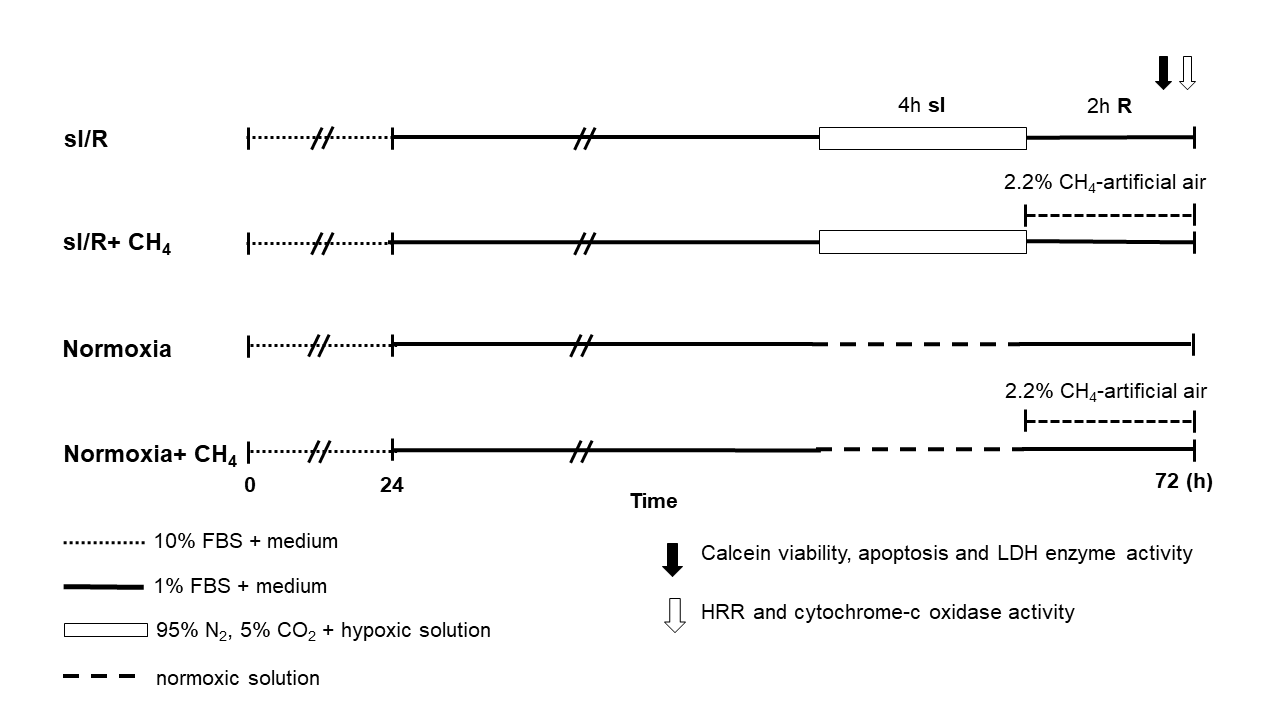

Supplement: Supplementary file 1 — Fig S1 [file JCMM-25-5113-s004.tif]

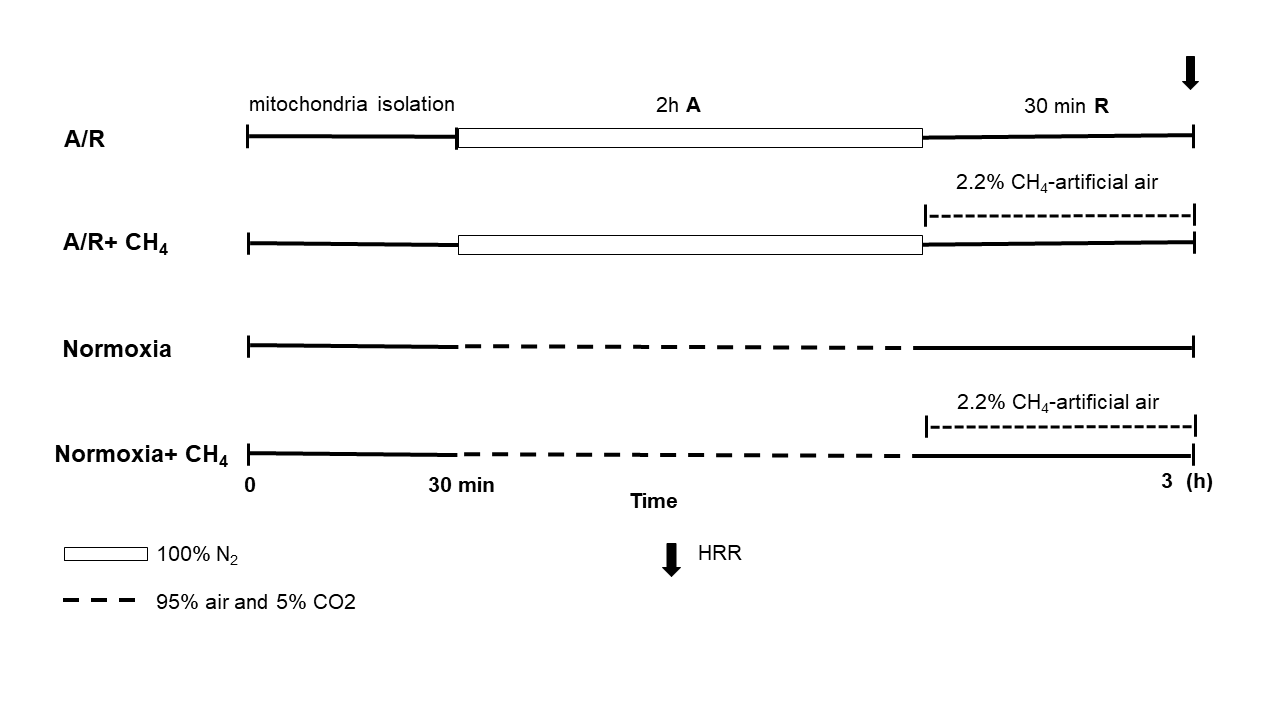

Supplement: Supplementary file 2 — Fig S2 [file JCMM-25-5113-s002.tif]

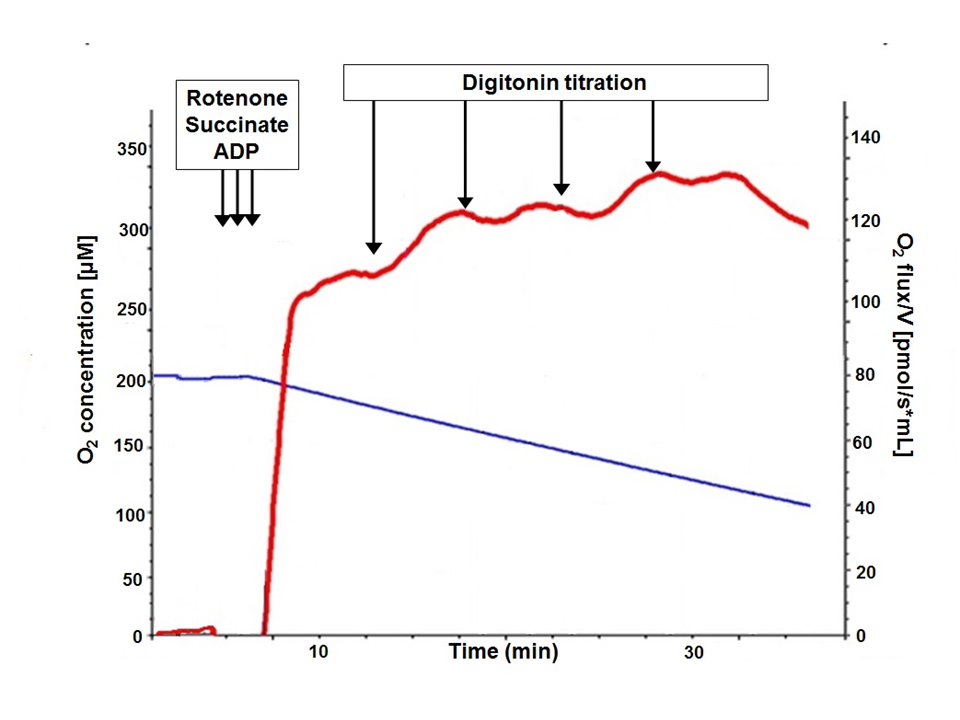

Supplement: Supplementary file 3 — Fig S3 [file JCMM-25-5113-s001.tif]

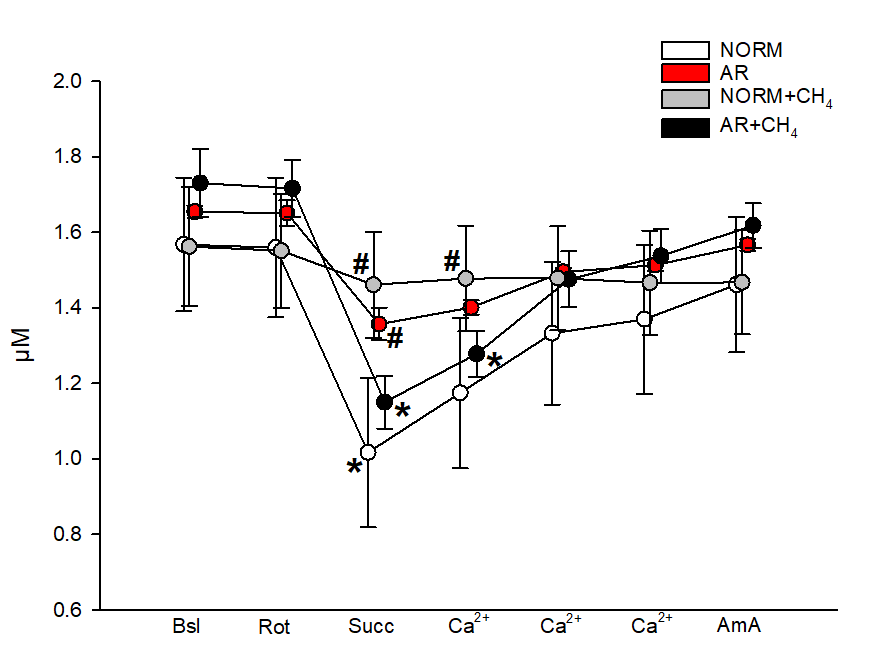

Supplement: Supplementary file 4 — Fig S4 [file JCMM-25-5113-s003.tif]
